# Generation of iPSCs from Jaw Periosteal Cells Using Self-Replicating RNA

**DOI:** 10.3390/ijms20071648

**Published:** 2019-04-03

**Authors:** Felix Umrath, Heidrun Steinle, Marbod Weber, Hans-Peter Wendel, Siegmar Reinert, Dorothea Alexander, Meltem Avci-Adali

**Affiliations:** 1 Department of Oral and Maxillofacial Surgery, University Hospital Tübingen, 72076 Tübingen, Germany; felix.umrath@med.uni-tuebingen.de (F.U.); siegmar.reinert@med.uni-tuebingen.de (S.R.); Dorothea.Alexander@med.uni-tuebingen.de (D.A.); 2 Department of Thoracic and Cardiovascular Surgery, University Hospital Tübingen, 72076 Tübingen, Germany; heidrun.steinle@uni-tuebingen.de (H.S.); marbod.weber@uni-tuebingen.de (M.W.); hans-peter.wendel@med.uni-tuebingen.de (H.-P.W.)

**Keywords:** induced pluripotent stem cells (iPSCs), jaw periosteal cells (JPCs), self-replicating RNA, reprogramming, iPSC-derived mesenchymal stem/stromal like cells (iMSCs), bone-tissue engineering

## Abstract

Jaw periosteal cells (JPCs) represent a suitable stem cell source for bone tissue engineering (BTE) applications. However, challenges associated with limited cell numbers, stressful cell sorting, or the occurrence of cell senescence during in vitro passaging and the associated insufficient osteogenic potential in vitro of JPCs and other mesenchymal stem/stromal cells (MSCs) are main hurdles and still need to be solved. In this study, for the first time, induced pluripotent stem cells (iPSCs) were generated from human JPCs to open up a new source of stem cells for BTE. For this purpose, a non-integrating self-replicating RNA (srRNA) encoding reprogramming factors and green fluorescent protein (GFP) as a reporter was used to obtain JPC-iPSCs with a feeder- and xeno-free reprogramming protocol to meet the highest safety standards for future clinical applications. Furthermore, to analyze the potential of these iPSCs as a source of osteogenic progenitor cells, JPC-iPSCs were differentiated into iPSC-derived mesenchymal stem/stromal like cells (iMSCs) and further differentiated to the osteogenic lineage under xeno-free conditions. The produced iMSCs displayed MSC marker expression and morphology as well as strong mineralization during osteogenic differentiation.

## 1. Introduction

Depending on the diagnosis, the perpetual challenge for clinicians in oral and maxillofacial surgery is to reconstruct and regenerate small as well as large bone defects. In previous studies, we and others demonstrated that human jaw periosteal cells (JPCs) can be used for the generation of bone tissue engineering (BTE) constructs [1,2,3]. For the regeneration of small defects, the use of the patient’s own jaw periosteal cells can be appropriate and the isolated cell numbers sufficient. The reconstruction of large bone resections with BTE products, e.g., for tumor patients, requires extremely high cell numbers. Unfortunately, the production of adequate cell yields is hampered by the occurrence of cell senescence and a decreased osteogenic potential of periosteal stem cells at higher passages. Additionally, primary JPCs, as well as mesenchymal stem/stromal cells (MSCs) from other tissues exhibit donor variability concerning their differentiation potential. This variability can be caused by different factors, such as varying stem cell numbers in heterogeneous starting materials, the tissue of origin, or the isolation and maintenance techniques [4,5]. Although surface markers have been identified, which allow the enrichment of osteogenic progenitor cells from heterogeneous cell populations via fluorescence or magnetic activated cell sorting (FACS, MACS) [6,7,8], cell yields are often unsatisfactory because of low sorting efficiencies and high cell mortality. 

The use of patient-specific induced pluripotent stem cells (iPSCs) could solve the above-mentioned problems. iPSCs can differentiate into every cell type of the body and their self-renewal capacity allows the expansion of desired cells to extremely high cell numbers [9]. They can be differentiated into mesenchymal stem/stromal like cells (iMSCs), which can serve as an alternative stem cell source for BTE [10]. Additionally, the directed differentiation of iPSCs to iMSCs could result in more homogeneous populations of osteogenic progenitor cells and therefore might help to standardize the starting material for clinical BTE products [11].

For the safe clinical applicability of iPSC-derived cells, the use of reprogramming methods excluding insertional mutagenesis and xeno-contaminations is of crucial importance [12]. Insertional mutagenesis can be prevented by reprogramming cells using synthetic mRNA, which is a safe and efficient reprogramming technique [13]. However, the transient nature of synthetic mRNAs requires daily transfections, which decrease the cell viability and make the process laborious and time-consuming. Yoshioka et al. [14] improved this approach by developing a synthetic polycistronic self-replicating RNA (srRNA) consisting of the OSKM reprogramming factors (OCT4, SOX2, KLF4, cMYC) and the non-structural proteins (nsP1 to nsP4) encoding sequences of the Venezuelan equine encephalitis virus (VEE), which enabled the RNA to replicate inside the transfected cells as long as their innate immune response was suppressed by the use of the interferon inhibitor B18R [14]. Using this srRNA, a single transfection can be sufficient to reprogram cells, and the removal of B18R leads to the degradation of the srRNA and results in the generation of footprint-free iPSCs. 

In the present study, we used an optimized srRNA construct containing a green fluorescent protein (GFP) encoding sequence to enable the live monitoring of transfected cells and the adaptation of the reprogramming protocol to the cell types used. For the production of clinically applicable BTE constructs, animal-derived components have to be excluded from every part of the generation procedure. Thus, in this study, we established for the first time a continuous xeno-free workflow from the initial primary JPC isolation to iPSC generation and differentiation into iMSCs until the final stage of mineralized tissue.

## 2. Results

### 2.1. srRNA Transfection Efficiency

**Figure 1:**
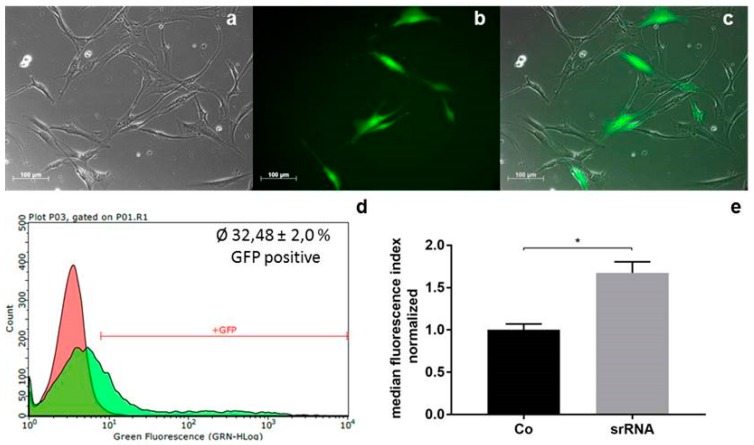
Transfection of jaw periosteal cells (JPCs) with self-replicating RNA (srRNA). (**a**–**c**) Representative (**a**) brightfield, (**b**) green fluorescent protein (GFP), and (**c**) merged images of JPCs 24 h after srRNA transfection. (**d**) Representative histogram of flow cytometry measurements of untransfected (red) and srRNA transfected (green) JPCs 24 h after transfection. (**e**) Average normalized median fluorescence index (MFI) values + standard deviation (SD) of srRNA transfected and untransfected (Co) JPCs relative to MFI values of untransfected (Co) samples were calculated and compared using Student’s *t*-test (*n* = 3, * *p* < 0.05).

To generate iPSCs, JPCs were seeded into 12-well plates at a density of 2.5 × 10^4^ cells/well and transfected with 0.25 ng of srRNA. After 24 h, transfected cells expressing GFP were detected by fluorescence microscopy (Figure 1a–c). An average transfection efficiency of 33 ± 2% was determined by the measurement of GFP-positive cells via flow cytometry (Figure 1d). Significantly higher (1.67-fold) median fluorescence index (MFI) values were detected in srRNA transfected JPCs compared to untransfected JPCs (Figure 1e).

### 2.2. JPC Reprogramming

**Figure 2:**
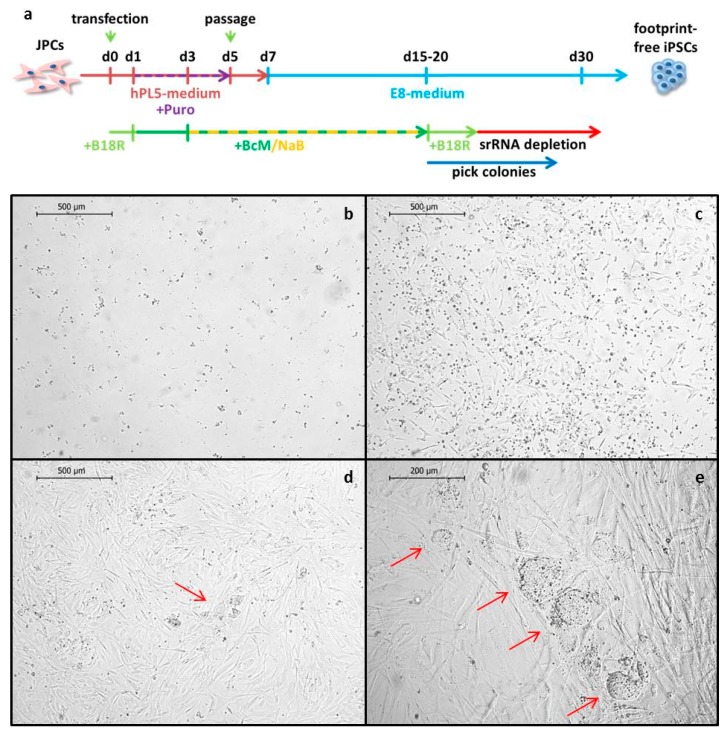
Reprogramming of JPCs. (**a**) Timeline of JPC reprogramming. JPCs were transfected (d0) in hPL5-medium containing 0.2 µg/mL B18R protein. On day one, the medium was changed to hPL5 containing 25% conditioned medium containing B18R (BcM) and 1.0 µg/mL puromycin (Puro). Puromycin selection was continued until day five (purple arrow). Cells were passaged on day five, and on day seven, the medium was changed to Essential 8 (E8) containing 25% BcM. Sodium butyrate (NaB) was added to the medium from day three to 15. When the first induced pluripotent stem cell (iPSC) colonies emerged, the medium was changed to E8 containing 0.2 µg/mL B18R protein. iPSC colonies were picked at day 15 or later. (**b**–**e**) Representative bright field images of JPCs during srRNA-based reprogramming. (**b**) Untransfected JPCs treated with puromycin at day five. (**c**) srRNA transfected JPCs treated with puromycin at day five. (**d**) srRNA transfected JPCs at day 12 with the first iPSC-colonies (indicated by a red arrow). (**e**) srRNA transfected cells at day 15 with iPSC-colonies (indicated by red arrows).

The reprogramming was performed with JPCs from three different patients according to Figure 2a. To select the srRNA containing cells 24 h after the transfection, 1 µg/mL puromycin was added to the medium. Furthermore, to enhance reprogramming efficiency, 250 µM sodium butyrate (NaB), which is a histone deacetylase inhibitor, was added to the medium. Puromycin selection was continued until day five, when all cells in the untransfected control wells were dead (Figure 2b). In comparison, Figure 2c shows the surviving cells transfected with srRNA at day five. After puromycin selection, cells were passaged and seeded in 1:5 ratio into vitronectin coated wells of 12-well plates. The first iPSC colonies emerged between day 12 and 15 (Figure 2d,e). Single iPSC colonies were picked and transferred into vitronectin coated 12-well plates containing E8 medium with 10 µM Y27632 ROCK inhibitor and were maintained in E8 medium without Y27632 from the next day onward. 

### 2.3. Characterization of iPSCs

#### 2.3.1. Pluripotency Marker Expression

**Figure 3:**
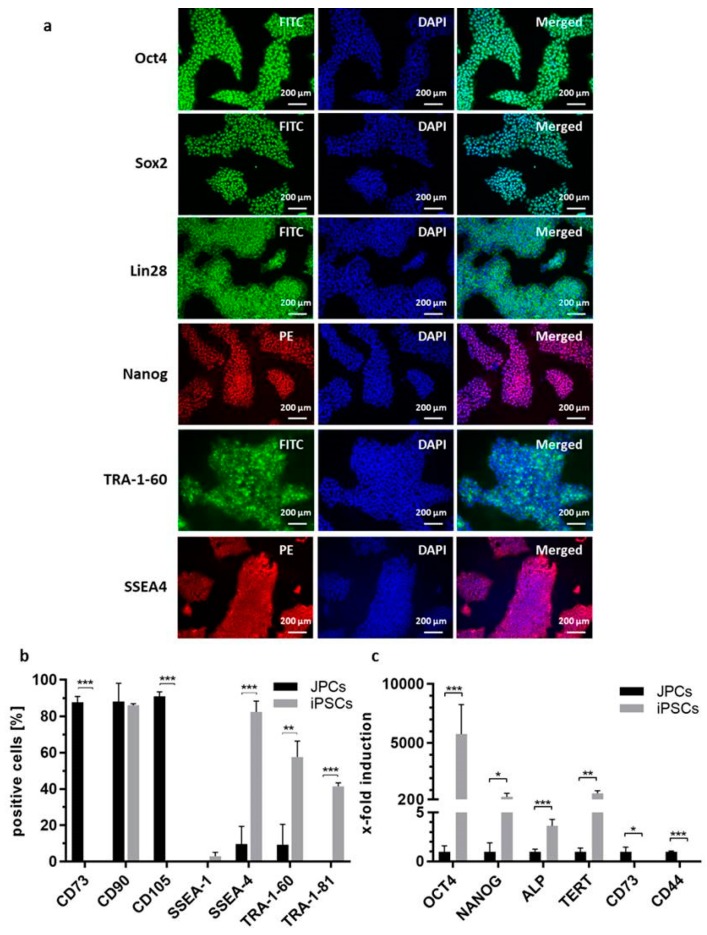
Pluripotency marker expression of JPC-derived iPSCs. (**a**) Oct4, Sox2, Lin28, Nanog, TRA-1-60 and SSEA4 immunostaining of iPSCs. (**b**) Surface marker expression of JPCs and JPC-derived iPSCs analyzed by flow cytometry and compared using Student’s *t*-test (*n* = 3, ** *p* < 0.01, *** *p* < 0.001). (**c**) Gene expression analysis of JPCs and JPC-derived iPSCs by qRT-PCR. Gene expression levels were normalized to levels of GAPDH. Mean values + SD of iPSC and JPC gene expression were displayed relative to those of JPCs. Statistical significance was calculated using Student’s *t*-test. (*n* = 3, * *p* < 0.05, ** *p* < 0.01, *** *p* < 0.001).

To characterize the generated iPSC clones, the expression of pluripotency markers was analyzed via immunostaining and flow cytometry. Figure 3a shows the strong fluorescence staining of Oct4, Sox2, Lin28, Nanog, TRA-1-60 and SSEA4 across all cells of the stained iPSC colonies. Furthermore, the surface marker expression of the generated iPSCs was compared to the initial JPCs using flow cytometry (Figure 3b). In the obtained iPSCs, the expression of MSC markers CD73 and CD105 was significantly downregulated, while pluripotent stem cell markers SSEA-4, TRA-1-60, and TRA-1-81 were significantly upregulated. JPCs and iPSCs were both positive for CD90 and negative for SSEA-1, as expected. Furthermore, the expression of MSC specific (CD73, CD44) and pluripotent stem cell specific (OCT4, NANOG, ALP, TERT) transcripts were analyzed by qRT-PCR. A significantly increased expression of pluripotent stem cell markers OCT4, NANOG, ALP, and TERT was detected in iPSCs compared to JPCs, while the expression of MSC specific transcripts CD73 and CD44 was significantly downregulated (Figure 3c).

#### 2.3.2. Differentiation Potential of iPSCs into the Three Germ Layers In Vitro and In Vivo 

**Figure 4:**
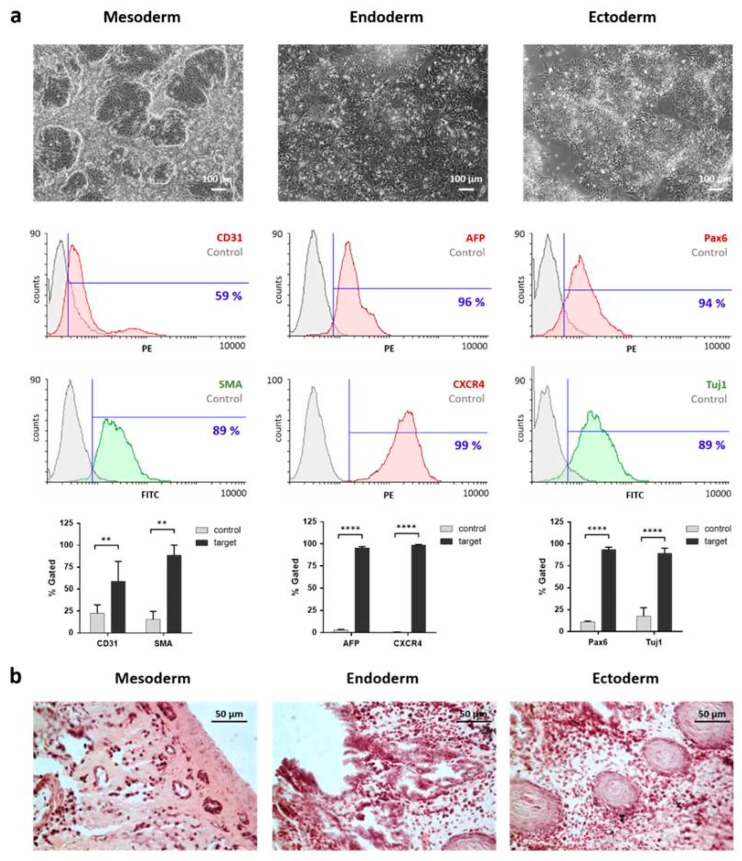
Trilineage differentiation potential of JPC-derived iPSCs. (**a**) Microscopic pictures of mesodermal, endodermal and ectodermal in vitro differentiation showing different morphologies after six days of germ layer specific differentiation. Flow cytometry analysis of differentiated cells was performed after staining with specific antibodies compared to untreated controls. Data are shown as mean + SD. Differences were compared using one-way ANOVA (*n* = 3, ** *p* < 0.01, **** *p* < 0.0001). (**b**) Microscopic images of in vivo teratoma formation of iPSCs using a chorioallantoic membrane (CAM) assay. H&E stained sections showed mesodermal (bone-like), endodermal (gut-like) and ectodermal (squamous epithelium) tissue.

The differentiation capacity of the generated iPSCs to form all tissues of the three germ layers is characteristic for pluripotent stem cells and was first assessed in vitro by a 7-day trilineage differentiation protocol. As shown in Figure 4a, endothelial, hepatocyte-like and neural-like cells were obtained after the differentiation of iPSCs. The mesoderm, endoderm, and ectoderm differentiation potential was confirmed by tissue specific antibody staining and quantification using flow cytometry. Mesodermal differentiation resulted in 59 ± 23% CD31-positive and 89 ± 11% SMA-positive cells. Endodermal induction yielded 96 ± 2% AFP-positive and 99 ± 1% CXCR4-positive cells. Ectodermal differentiation was demonstrated by the detection of 94 ± 3% Pax6-positive and 89 ± 6% Tuj1-positive cells.

Using a chicken embryo chorioallantoic membrane (CAM) assay, the in vivo differentiation of iPSCs was analyzed. 10 days after the application of iPSCs onto the CAM, teratoma formation could be observed. Subsequently, teratomas were sectioned and stained with hematoxylin & eosin (H&E) and tissue types of the mesodermal (bone-like tissue), endodermal (gut-like tissue) and ectodermal (squamous epithelium) lineage could be identified (Figure 4b).

#### 2.3.3. Detection of srRNA in iPSCs and Karyotyping of iPSCs

**Figure 5:**
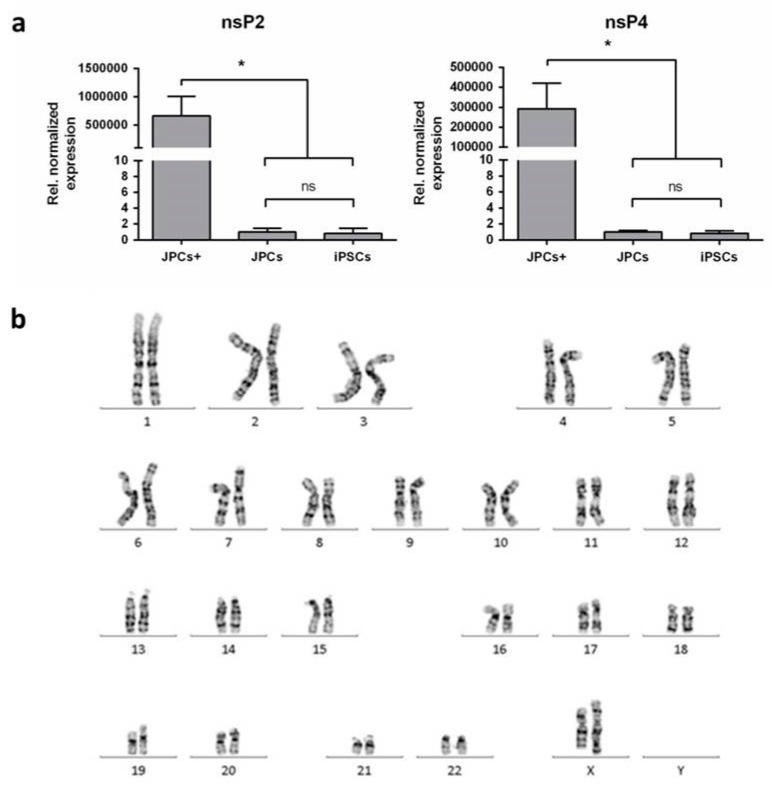
Elimination of srRNA after iPSC generation and karyotyping of JPC-derived iPSCs. (**a**) qRT-PCR analysis of nsP2 and nsP4 transcripts in iPSCs (passage 3), untreated JPCs and srRNA containing JPCs (JPCs+) 48 h post-transfection. Data are shown as mean + SEM. Differences were compared using one-way ANOVA (*n* = 3, * *p* < 0.05, ns = not significant) (**b**) Representative karyogram of JPC-derived iPSCs showing a normal karyotype (46, XX).

After the successful generation of iPSCs, the presence of srRNA in the cells was analyzed. Therefore, qRT-PCR was performed with RNA isolated from JPCs and JPC-derived iPSCs and primer pairs specific to the nsP2 and nsP4 sequences. JPCs transfected with srRNA and cultivated for 48 h, served as positive control (JPC+). These samples showed high amounts of nsP2 and nsP4 compared to untransfected JPCs and iPSCs (Figure 5a). In contrast, the nsP2 and nsP4 amount measured in iPSCs was similar to that of untransfected JPCs, which demonstrates the absence of srRNA in the reprogrammed cells. Karyotyping of JPC-derived iPSCs resulted in normal karyograms without chromosomal aberrations (Figure 5b).

#### 2.3.4. Differentiation of iPSCs into iMSCs and Their Characterization

**Figure 6:**
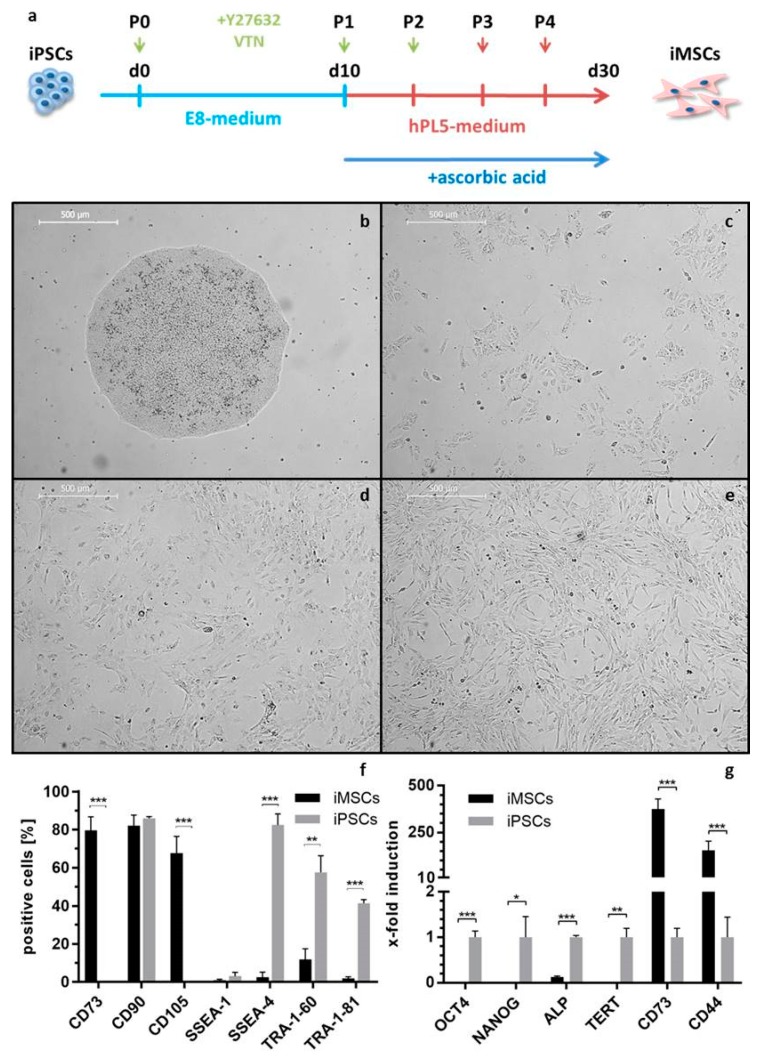
Differentiation of iPSCs into iPSC-derived mesenchymal stem/stromal like cells (iMSCs). (**a**) Timeline: iPSCs were seeded onto vitronectin coated plates in the presence of the ROCK inhibitor Y27632 and cultivated for 10 days in E8 medium (light blue line). Thereafter, human platelet lysate hPL5 medium containing 150 µM ascorbic acid was added to the cells (red line). Cells were passaged 3–5 times until they showed homogeneous MSC-like morphology after approximately 30 days. (**b**–**e**) Change of morphology during the differentiation of iPSCs into iMSCs. (**b**) iPSC colony before differentiation. (**c**) Cells after single cell plating in passage 0. (**d**) Differentiating iMSCs in passage two and (**e**) iMSCs in passage four (scale bars represent 500 µm). (**f**) Surface marker expression of iMSCs compared to iPSCs detected by flow cytometry. (**g**) Gene expression levels of iMSCs were normalized to levels of the housekeeping gene glyceraldehyde 3-phosphate dehydrogenase (GAPDH) and presented as x-fold induction relative to iPSCs (set to 1). Differences in surface marker, and gene expression were compared using Student’s *t*-test. (*n* = 3, * *p* < 0.05, ** *p* < 0.01, *** *p* < 0.001).

To be able to use iPSCs for BTE, the differentiation potential of iPSCs into the osteogenic lineage has to be demonstrated. Therefore, iPSCs were first differentiated into iMSCs (Figure 6a). For this purpose, iPSCs (Figure 6b) were cultivated without passaging for 10 days to stimulate spontaneous differentiation. Subsequently, cells were passaged as single cells and incubated in hPL5 medium with ascorbic acid until their morphology changed to a spindle shaped MSC-like appearance (3–5 passages, Figure 6c,d). Cells exhibiting MSC morphology (Figure 6e) were expanded in hPL5 medium before osteogenic differentiation and characterization. 

Using flow cytometry, the surface marker expression of obtained iMSCs was analyzed (Figure 6f). The expression of MSC markers (CD73 and CD105) was significantly upregulated in iMSCs, while iPSC markers (SSEA-4, TRA-1-60, and TRA-1-81) were significantly downregulated compared to iPSCs. iMSCs and iPSCs were both positive for CD90 and negative for SSEA-1 (Figure 6f). In addition, gene expression of MSC markers (CD73, CD44) and iPSC markers (OCT4, NANOG, ALP, and TERT) was quantified by qRT-PCR (Figure 6g). iPSC markers were significantly downregulated in iMSCs compared to iPSCs, while MSC markers were significantly upregulated.

#### 2.3.5. Osteogenic Differentiation of iMSCs

**Figure 7:**
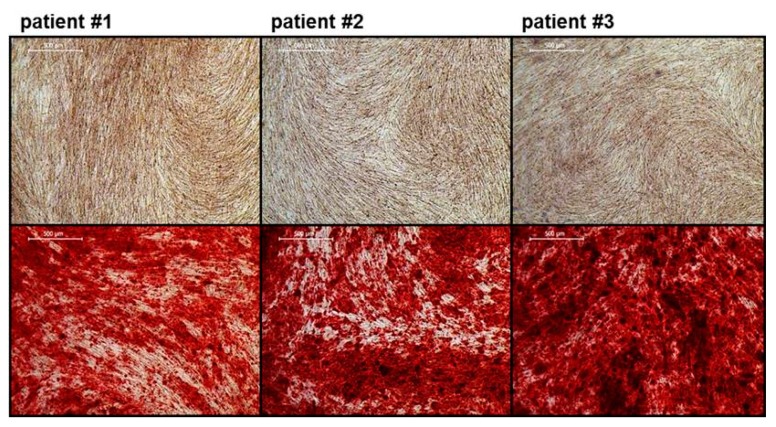
Osteogenic differentiation potential of iMSCs. iMSCs incubated without osteogenic stimuli (control, upper panel) and osteogenic medium (lower panel) for 15–20 days, stained with alizarin red (scale bars represent 500 µm).

To demonstrate the functionality of iMSCs and their potential to be used for BTE applications, iMSCs were subjected to osteogenic differentiation. To differentiate the iMSCs under xeno-free conditions, cells were treated with osteogenic medium supplemented with human platelet lysate (hPL) instead of fetal bovine serum (FBS). After 15–20 days of differentiation, cells were fixed, and calcium phosphate precipitation was stained with alizarin red. As displayed in Figure 7, all iMSCs were able to produce strong mineral deposits.

## 3. Discussion

Jaw periosteum is an excellent, but limited source of osteogenic progenitor cells. To establish stem cell based regenerative therapies for large bone defects in oral and maxillofacial surgeries, appropriate numbers of good quality stem cells are needed. However, it is difficult to obtain sufficient cell numbers from cell cultures expanded from primary cells. The occurrence of cell senescence and the loss of differentiation potential are problems to be solved. Thus, iPSCs are a promising alternative cell source which might help to solve these problems. Unfortunately, the use of iPSC derived cells still has some safety issues concerning insertional mutagenesis caused by reprogramming vectors, teratoma formation, and infections or immune responses caused by xenogenic media supplements. In our study, we established protocols to overcome these issues and to bring therapeutic applications of iPSC-derived osteogenic progenitor cells closer to the clinic. To generate footprint-free iPSCs and to address the problem of insertional mutagenesis, srRNA was used, which combines the safety of synthetic mRNA-based reprogramming and the convenience of a single transfection.

The complete removal of srRNA after B18R withdrawal was demonstrated in the generated iPSCs at passage three, as also shown by Yoshioka et al. [14]. The additional presence of a GFP encoding sequence in the srRNA allowed convenient monitoring of srRNA translation and depletion following termination of immunosuppression by B18R interferon inhibitor. Thus, it allowed the adaptation of reprogramming/transfection protocols for JPCs. The reprogramming protocol established in this study worked reliably for JPCs from all tested patients and could also be successfully applied to human gingival fibroblasts (data not shown). 

The osteogenic differentiation of iPSCs can be performed with different protocols, however recently a two-step approach was described, where iPSCs were first differentiated iMSCs, and in a second step subjected to osteogenic differentiation [10,15]. The differentiation of iPSCs into iMSCs has several advantages, e.g., the exclusion of teratoma formation risk [16] and the possibility to use cell culture and differentiation protocols standardized for MSCs. Usually, the differentiation of iPSCs to iMSCs can either be performed via embryoid body formation or by the incubation of iPSCs (when growing as colonies or plated as single cells) with differentiation media, followed by several passaging steps [17]. 

In this study, we modified a protocol from Luzzani et al. (2015), who also used hPL containing media for the differentiation of iPSCs into iMSCs [18]. To improve the yield of iMSCs, iPSC colonies were cultivated for extended periods without passaging prior to single cell plating. The resulting high cell densities probably primed the cells towards mesenchymal differentiation.

As shown by flow cytometry and gene expression analysis, the surface marker and gene expression profile of the obtained iMSCs were similar to that of typical MSCs [4]. Subsequent osteogenic differentiation resulted in strong mineral deposition, which demonstrated the promising potential of these cells for BTE applications.

In the present work, an important step towards clinical application was made by removing all xenogenic compounds from the protocols throughout the process. This was possible by replacing FBS with hPL for medium supplementation. Our attempts to generate iPSCs from JPCs using commercially available defined MSC-media failed due to low cell viability after the srRNA transfection or puromycin selection. 

The translation of iPSCs from basic research to clinical application has made substantial progress in the past years. The first clinical trial using iPSCs to treat macular degeneration has been launched in Japan in 2014, and was able to demonstrate the safety of iPSC-derived regenerative therapies [19]. Further, a clinical trial using iPSC-derived MSCs for the treatment of steroid-resistant acute graft versus host disease (GvHD) has been started in Australia in March 2017 [20]. Preliminary results of this trial also proved the safety of the iPSC-derived MSCs and showed an improvement in severity of GvHD in 14 out of 15 patients. 

These trials raise hopes for other iPSC applications to reach the clinical level in the near future even though it is still a long way for iPSCs to make their way into clinical routine.

## 4. Materials and Methods 

### 4.1. Xeno-Free Isolation and Culture of JPCs

JPCs derived from three patients were included in this study in accordance with the local ethical committee (approval number 074/2016BO2, 17.05.2016) and after obtaining written informed consent. Jaw periosteal tissue was cut in small pieces with a scalpel and incubated in DMEM/F12 (Thermo Fisher Scientific, Waltham, MA, USA) containing 10% hPL (ZKT Tübingen gemeinnützige GmbH), 100 U/mL penicillin-streptomycin (Lonza, Basel, Switzerland), 2.5 µg/mL amphotericin B (Biochrom, Berlin, Germany), 50 µg/mL gentamicin (Lonza), and 10 µg/mL ciprofloxacin (Sigma-Aldrich, St. Louis, USA) for 1–2 weeks. Outgrowing cells were passaged using TrypLE Express (Thermo Fisher Scientific) and expanded and frozen in passage one using Cryo SFM freezing medium (Promocell, Heidelberg, Germany). From passage two onward, JPCs were grown in hPL5-medium (DMEM/F12 containing 5% hPL, 100 U/mL penicillin-streptomycin, and 2.5 µg/mL amphotericin B).

### 4.2. Production of srRNA

The T7-VEE-OKS-iM plasmid, a gift from Steven Dowdy (Addgene plasmid # 58972 ), containing sequences encoding the non-structural proteins (nsP1 to nsP4) for self-replication, the reprogramming factors Oct4, Klf4, Sox2, and cMyc [14] and an additionally added internal ribosome entry site (IRES)-GFP was amplified in *E.coli* and plasmids were isolated using QIAPrep (Qiagen, Hilden, Germany). After the linearization with MluI restriction enzyme (Thermo Fisher Scientific), 10 µg template DNA was transcribed in vitro using RiboMAX large-scale production system T7 Kit (Promega, Madison, WI, USA) according to the manufacturer’s instructions. Afterwards, 2 U TURBO DNase was added for 15 min at 37 °C. For 5′-end capping, ScriptCap Cap1 Capping System was used followed by 3′-end polyadenylation with A-Plus Poly(A) Polymerase Tailing Kit (both from Cellscript, Madison, WI, USA) according to the manufacturer’s instructions. Following each reaction step, srRNA was purified using RNeasy Kit (Qiagen). The specific lengths of the generated DNA and srRNA products were analyzed using 1% agarose gel electrophoresis.

### 4.3. Production of B18R-mRNA 

The coding sequence for B18R was inserted by Aldevron (Fargo, ND, USA) into the pcDNA 3.3 plasmid. Pseudoruridine-5′-triphosphate (Ψ-UTP) and 5-methylcytidine-5′-triphosphate (m5CTP) modified B18R mRNA was generated by in vitro transcription (IVT) as previously described in our studies [21].

### 4.4. Generation of Integration-Free iPSCs from JPCs Using srRNA

#### 4.4.1. Preparation of Conditioned Medium Containing B18R (BcM)

JPCs were expanded in hPL5 medium and passaged into a T75 flask to reach approx. 80% confluency at the next day. To perform B18R-mRNA transfection, the medium was aspirated and replaced by 6.5 mL Opti-MEM (Thermo Fisher Scientific). The transfection cocktail was prepared according to the manufacturer’s instructions (500 µL Opti-MEM, 7.5 µg of B18R-mRNA, 15 µL Lipofectamine 3000 (Thermo Fisher Scientific)), added to the medium and incubated for 4 h at 37 °C. Subsequently, medium was changed to 15 mL hPL5 medium. After 24 h, medium was collected and replaced with 15 mL fresh hPL5. The collected BcM was stored at −20 °C. Medium collection was repeated until day three and the collected BcM was pooled, sterile filtered with a 0.2 µm filter, aliquoted and stored at −20 °C.

#### 4.4.2. Transfection of JPCs with srRNA and Reprogramming

JPCs (2.5–5 × 10^4^ cells) were seeded into 12-well plates to reach 30–50% confluency the next day. Medium was changed to 0.5 mL hPL5 supplemented with 0.2 µg/mL recombinant B18R protein (eBioscience, San Diego, CA, USA) 30 min prior to transfection. The transfection cocktail (25 µL Opti-MEM, 0.25 µg srRNA, 0.5 µL TransIT mRNA Boost Reagent (Mirus Bio LLC, Madison, WI, USA), and 0.5 µl TransIT mRNA Reagent (Mirus Bio LLC)) was prepared according to the manufacturer’s instructions and added to the wells.

24 h after transfection, the medium was replaced with hPL5 containing 25% BcM and 1 µg/mL puromycin (Thermo Fisher Scientific) and changed every other day. To determine the transfection efficiency, cells were harvested after 24 h using TrypLE Express, resuspended in BD Cytofix/Cytoperm Solution (BD Bioscience, Franklin Lakes, NJ, USA) and GFP expression was measured by flow cytometry using the Guava EasyCyte 6HT-2L instrument (Merck Millipore, Billerica, MA, USA). From day three until day 15, 250 µM sodium butyrate (NaB, Selleck Chemicals LLC, Houston, TX, USA) was added to the medium. On day five, after successful puromycin selection, srRNA containing cells were passaged using TrypLE Express and split in 1:5 ratio into 12-well plates coated with 0.5 mL of a 5 µg/mL vitronectin solution (Thermo Fisher Scientific). Untransfected cells died within the five days of puromycin treatment. Two days after passaging (day seven), the medium was changed to Essential 8 medium (E8, Thermo Fisher Scientific) containing 25% BcM and replaced by fresh medium every day. When first iPSC colonies emerged, medium was changed to E8-medium supplemented with 0.2 µg/mL recombinant B18R protein. Single iPSC colonies were picked and transferred into vitronectin coated 12-well plates containing E8 medium supplemented with 10 µM Y27632 ROCK inhibitor (Selleck Chemicals LLC). iPSCs were maintained in E8 medium with daily medium changes and passaged every 4–6 days.

### 4.5. Characterization of JPC-Derived iPSCs

#### 4.5.1. Immunostaining for Detection of Pluripotency 

On vitronectin coated glass slides, 5 × 10^5^ iPSCs were cultivated for 2–3 days and fixed using fixation solution (R&D Systems, Minneapolis, MN, USA) for 10 min. Afterwards, cells were washed with wash buffer (Permeabilization/Wash Buffer I, R&D Systems) and blocked with 5% BSA in wash buffer for 1–2 h at RT. Antibody staining was performed according to the manufacturer’s instructions in wash buffer containing 1% BSA. Cells were incubated for 3 h at RT with fluorescently labelled antibodies (PE-labelled mouse anti-human Nanog antibody (BD Bioscience), DyLight™ 488 labelled mouse anti-human StainAlive™ TRA-1-60 antibody (Stemgent, Cambridge, MA, USA), and DyLight™ 550 labelled mouse anti-human StainAlive™ SSEA-4 antibody (Stemgent)). The incubation with primary antibodies (rabbit anti-human POU5F1 (Oct4) (Sigma-Aldrich), rabbit anti-human Sox2 (Stemgent), and mouse anti-human LIN28A (6D1F9) (Thermo Fisher Scientific)) were performed overnight at 4°C. After washing with wash buffer, cells were incubated for 1 h in wash buffer containing 1% BSA with fluorescently labeled secondary antibodies (FITC-labelled sheep anti-mouse IgG (Sigma-Aldrich) or Cy3-labelled goat anti-rabbit IgG (Thermo Fisher Scientific)). Finally, the cells were washed and stained using Fluoroshield mounting medium with DAPI (Abcam, Cambridge, UK). Fluorescence microscopic images were taken using Axiovert135 microscope and AxioVision 4.8.2 software (Carl Zeiss, Oberkochen, Germany).

#### 4.5.2. Tri-Lineage Differentiation of iPSCs In Vitro

The ability of iPSCs to differentiate into the three germ layers was analyzed using the human StemMACS™ Trilineage Differentiation Kit (Miltenyi, Bergisch Gladbach, Germany) according the manufacturer’s instructions. 1 × 10^5^ iPSCs were seeded per well of a 12-well plate for mesoderm differentiation, 2 × 10^5^ cells for endoderm differentiation, and 1.5 × 10^5^ iPSCs for ectoderm differentiation. The cells were analyzed after seven days of differentiation using flow cytometry. 

Therefore, cells were detached, fixed for 10 min at RT in fixation solution (R&D Systems) and washed with DPBS. Then, the cells were incubated for 45 min at RT with wash buffer (Wash Buffer I, R&D Systems) containing 5 µl of each of the following antibodies: Mesoderm differentiation: Alexa Fluor 488 labelled anti-human α-smooth muscle actin (α-SMA) antibody (R&D Systems) and PE labelled mouse anti-human CD31 antibody (BD Biosciences), Endoderm differentiation: PE-labelled anti-human C-X-C chemokine receptor type 4 (CXCR4) antibody and PE-labelled anti-human α-fetoprotein (AFP) antibody (both from R&D Systems, Minneapolis, USA), Ectoderm differentiation: Alexa Fluor 488 labelled anti-human neuron-specific class III β-tubulin (Tuj1) antibody (BD Biosciences) and PE-labelled anti-human paired box gene 6 (Pax6) antibody (Miltenyi). After washing with wash buffer, cells were fixed using CellFIX solution (BD Biosciences) and analyzed using FACScan flow cytometer (BD Biosciences) and Flowing Software (Turku Centre for Biotechnology, Turku, Finland).

#### 4.5.3. Teratoma Formation on Chicken Embryo Chorioallantoic Membrane (CAM)

To confirm the differentiation potential of iPSCs into the three germ layers, an in vivo teratoma formation was performed using CAM assay. After three days of incubation at 37 °C, 2-3 mL albumen was aspirated from fertilized chicken eggs (Lohmann White x White Rock) and a window was cut into the shell and sealed with semi-permeable adhesive tape. After seven days of incubation at 37 °C, a silicone ring was placed onto the CAM and 2 × 10^6^ iPSCs mixed with 50 µL E8 medium and 50 µL Matrigel (Corning, New York, NY, USA) were transferred into the inner circle of the silicone ring. The eggs were then sealed again and further incubated at 37 °C. After 10 days (day 17 of incubation), the teratoma cell mass with the surrounding CAM was excised and fixed at 4 °C with 4% paraformaldehyde (Merck, Darmstadt, Germany) overnight. After washing with DPBS and ethanol dehydration, the samples were embedded in paraffin for sectioning and H&E (Morphisto, Frankfurt, Germany) staining.

#### 4.5.4. Detection of Residual srRNA in the Reprogrammed iPSCs

The presence of srRNA in the generated iPSCs was analyzed by qRT-PCR using primers specific for sequences encoding the non-structural proteins nsP2 and nsP4: nsP2 (fw: 5′-TCC ACA AAA GCA TCT CTC GCC G-3′, rev: 5′-TTT GCA ACT GCT TCA CCC ACC C-3′) and nsP4 (fw: 5′-TTT TCA AGC CCC AAG GTC GCA G-3′, rev: 5′-TGT TCT GGA TCG CTG AAG GCA C-3′). For RNA isolation, 1 × 10^6^ iPSCs at passage three were used (Aurum^TM^ Total RNA Mini Kit (Bio-Rad, Hercules, CA, USA) and 300 ng RNA was reverse transcribed into complementary DNA (cDNA) using iScript Kit (Bio-Rad). qRT-PCR reactions with 40 cycles were performed in CFX Connect Real-Time PCR Detection System (Bio-Rad) using IQ™ SYBR^®^ Green Supermix (Bio-Rad) and 300 nM primer with following conditions: 3 min at 95 °C (1 cycle); 95 °C for 15 s, 60 °C for 30 s, and 72 °C for 10 s. The mRNA expression levels were normalized to glyceraldehyde 3-phosphate dehydrogenase (GAPDH). The results are shown relative to the initial JPCs.

#### 4.5.5. Karyotyping of iPSCs

iPSCs were grown to 60–80% confluency in a 6-well plate and incubated for 90 min with 0.15 µg/mL KaryoMAX colcemid solution (Thermo Fisher Scientific). Then, cells were detached using Accutase (Thermo Fisher Scientific) and inactivated with DMEM/F12 containing 10% FBS. The cell suspension was centrifuged (300× *g* for 5 min), the supernatant was discarded and 1.5 mL of 0.075 M KaryoMAX KCl solution (Thermo Fisher Scientific) was added and incubated at 37 °C. After 30 min, 100 µL fixative (3:1 methanol/acetic acid) was added and incubated for 10 min. After centrifugation, the cells were resuspended in fixative, incubated for 1 h at RT, and then stored over night at 20 °C. Chromosome analysis was performed by the Institute of Medical Genetics and Applied Genomics of the University Hospital Tübingen.

### 4.6. Differention of iPSCs into iMSCs

Very low concentrations of iPSCs (≤ 10% confluency) were seeded into vitronectin coated (0.5 mL of 5 µg/mL solution) 12-well plates and cultivated without passaging for 10 days to stimulate spontaneous differentiation. After this period, the cells were detached using Accutase and transferred into vitronectin coated 6-well plates containing E8-medium and 10 µM ROCK inhibitor Y27632 (passage 1). The next day, the medium was changed to hPL5 supplemented with 150 µM L-ascorbic acid 2-phosphate (Sigma-Aldrich) and medium was replaced every other day. After reaching 80% confluency, cells were passaged into vitronectin coated 6-well plates (split ratio 1:3) using Accutase and ROCK inhibitor was added to the medium. For following cell passages TrypLE Express was used and no further ROCK inhibitor or vitronectin was used. Cells were passaged until the morphology of the cells had changed to a spindle shaped MSC-like appearance (3–5 passages). Cells exhibiting MSC morphology were expanded in hPL5 medium before using the cells for osteogenic differentiation and characterization.

### 4.7. Osteogenic Differentiation of iMSCs

For osteogenic differentiation, iMSCs were cultivated in osteogenic medium (DMEM/F12 containing 10% hPL, 100 U/mL penicillin-streptomycin (Lonza), 2.5 µg/mL amphotericin B, 0.1 mM l-ascorbic acid 2-phosphate (Sigma-Aldrich), β-glycerophosphate (AppliChem, Darmstadt, Germany), and 4 µM dexamethasone (Sigma-Aldrich)) with medium changes every 2–3 days. After 15–25 days, cells were fixed with 4% formalin and stained for 20 min with 1 mL of 40 mM Alizarin red solution (pH 4.2, Sigma-Aldrich). Unbound dye was removed by washing with deionized water and images were taken using an inverted microscope (Leica, Wetzlar, Germany).

### 4.8. Flow Cytometric Analysis of JPCs, iPSCs, and iMSCs

The expression of pluripotency markers (SSEA-1, SSEA-4, TRA-1-60, TRA-1-80) and MSC-markers (CD73, CD90, CD105) was analyzed by flow cytometry. Cells were detached using TrypLE Express and 1 × 10^5^ cells per sample were incubated on ice for 15 min in 20 µL blocking buffer (PBS, 0.1% BSA, 1 mg/mL sodium azide (Sigma-Aldrich), and 10% Gamunex (human immune globulin solution, Talecris Biotherapeutics GmbH, Frankfurt, Germany)). Then, 50 µL FACS buffer (PBS, 0.1% BSA, 1 mg/mL sodium azide) as well as phycoerythrin (PE) and allophycocyanin (APC) conjugated antibodies (for individual volumes see Table 1) were added and incubated on ice for 20 min. After two washing steps with 200 µL FACS buffer, flow cytometry measurements were performed using the Guava EasyCyte 6HT-2L (Merck Millipore, Billerica, MA, USA).

### 4.9. Gene expression Analysis of JPCs, iPSCs, and iMSCs by qRT-PCR 

RNA isolation from JPCs, iPSCs and iMSCs (1 × 10^5^ cells per sample) was carried out using the NucleoSpin RNA kit (Macherey-Nagel, Düren, Germany) following the manufacturer’s instructions. The amount of isolated RNA was quantified with a Qubit 3.0 fluorometer and the corresponding RNA BR Assay Kit (Thermo Fisher Scientific). The first-strand cDNA synthesis was performed with 0.5 μg of RNA using the SuperScript Vilo Kit (Thermo Fisher Scientific). The quantification of mRNA expression levels was performed using the real-time LightCycler System (Roche Diagnostics, Mannheim, Germany). For the PCR reactions, commercial OCT4, NANOG, ALP, CD44, and CD73 primer kits (Search LC, Heidelberg, Germany) and DNA Master SYBR Green I kit (Roche, Basel, Switzerland) were used. The amplification was performed with a touchdown PCR protocol of 40 cycles (annealing temperature between 68–58 °C), following the manufacturer’s instructions. GOI (gene of interest) transcript levels of each sample were normalized to those of the housekeeping gene GAPDH, divided by the corresponding control samples and displayed as x-fold induction indices.

### 4.10. Statistical Analysis

For the statistical evaluation of data, means + standard deviation (SD) or standard error of mean (SEM) were calculated. Student’s *t*-test or one-way analysis of variance (ANOVA) for repeated measurements followed by Bonferroni’s multiple comparison test was used. All statistical analyses were performed double-tailed using GraphPad Prism 6.01. A *p*-value < 0.05 was considered significant.

## 5. Conclusions

In this study, we generated for the first time, footprint- and xeno-free iPSCs from JPCs by the transfection of srRNA encoding the reprogramming factors. We conclude that JPCs can function as starting material for the generation of clinical grade autologous iPSCs. The differentiation of JPC-iPSCs to iMSCs leads to the generation of cells with a high osteogenic potential, which are a promising source of osteogenic progenitor cells for BTE. Using cGMP grade hPL as a medium supplement, clinically applicable osteogenic progenitor cells can be obtained.

## Figures and Tables

**Table 1:** List of antibodies used for flow cytometry.

Human Antigen	Volume per Sample (µL)	Isotype	Conjugate	Company
SSEA1	5	human recombinant antibody (REA)	PE	Miltenyi, Bergisch Gladbach, Germany
SSEA4	5		PE	
TRA-1-60	5		PE	
TRA-1-81	5		PE	
REA-Isotype	5		PE	
CD73	5	mouse IgG1	PE	BD Biosciences, Franklin Lakes, NJ, USA
CD90	1		PE	
CD105	10		APC	BioLegend, San Diego, CA, USA
IgG1-Isotype	10		APC	
IgG1-Isotype	5		PE	R&D Systems, Minneapolis, MN, USA

## Abbreviations

ANOVA	analysis of variance
APC	allophycocyanin
BcM	conditioned medium containing B18R
BSA	bovine serum albumin
BTE	bone tissue engineering
CAM	chorioallantoic membrane
cGMP	current good manufacturing practice
cDNA	complementary DNA
DNA	deoxyribonucleic acid
DPBS	Dulbecco's phosphate buffered saline
FACS	fluorescence-activated cell sorting
FBS	fetal bovine serum
GFP	green fluorescent protein
GOI	gene of interest
H&E	haematoxylin and eosin
hPL	human platelet lysate
IgG	immunoglobulin G
iMSC	iPSC-derived mesenchymal stem/stromal like cell
iPSC	induced pluripotent stem cell
IRES	internal ribosome entry site
JPC	jaw periosteal cell
m5CTP	5-methylcytidine-5′-triphosphate
MACS	magnetic-activated cell sorting
MFI	median fluorescence index
mRNA	messenger RNA
MSC	mesenchymal stem/stromal cell
NaB	sodium butyrate
OSKM	OKT4, SOX2, KLF4, cMYC
PBS	phosphate buffered saline
PCR	polymerase chain reaction
PE	phycoerythrin
Puro	puromycin
qRT-PCR	quantitative real-time polymerase chain reaction
RNA	ribonucleic acid
ROCK	rho-associated protein kinase
SD	standard deviation
SEM	standard error of mean
srRNA	self-replicating ribonucleic acid
Ψ-UTP	pseudoruridine-5′-triphosphate
